# Unraveling the Mysteries of Mental Illness With Psilocybin

**DOI:** 10.7759/cureus.25414

**Published:** 2022-05-27

**Authors:** Robert Sottile, Herpreet Singh, Anne Weisman, Thomas Vida

**Affiliations:** 1 Medical Education, Kirk Kerkorian School of Medicine at University of Nevada Las Vegas, Las Vegas, USA

**Keywords:** review of clinical trials, neuroplasticity, intrinsic brain networks, neuritogenesis, psychoactive drug, major depressive disorder (mdd), treatment-resistant depression, mental illness, psilocybin, psychedelics

## Abstract

Current medications have not been effective in reducing the prevalence of mental illness worldwide. The prevalence of illnesses such as treatment-resistant depression has increased despite the widespread use of a broad set of psychopharmaceuticals. Transcranial magnetic stimulation and ketamine therapy are making great strides in improving treatment-resistant depression outcomes but they have limitations. New psychotherapeutics are required that specifically target the underlying cellular pathologies leading to neuronal atrophy. This neuronal atrophy model is supplanting the long-held neurotransmitter deficit hypothesis to explain mental illness. Interest in psychedelics as therapeutic molecules to treat mental illness is experiencing a 21st-century reawakening that is on the cusp of a transformation. Psilocybin is a pro-drug, found in various naturally occurring mushrooms, that is dephosphorylated to produce psilocin, a classic tryptamine psychedelic functional as a 5-hydroxytryptamine 2A receptor agonist. We have focused this review to include studies in the last two years that suggest psilocybin promotes neuronal plasticity, which may lead to changes in brain network connectivity. Recent advancements in clinical trials using pure psilocybin in therapy suggest that it may effectively relieve the symptoms of depression in patients diagnosed with major depressive disorder and treatment-resistant depression. Sophisticated cellular and molecular experiments at the systems level have produced evidence that demonstrates psilocybin promotes neuritogenesis in the mouse brain - a mechanism that may address the root cause of depression at the cellular level. Finally, studies with psilocybin therapy for major depressive disorder suggest that this ancient molecule can promote functionally connected intrinsic networks in the human brain, resulting in durable improvements in the severity of depressive symptoms. Although further research is necessary, the prospect of using psilocybin for the treatment of mental illness is an enticing possibility.

## Introduction and background

The increasing prevalence of mental illness

A dire need exists for novel medications capable of treating mental illnesses with significantly higher efficacy than the pharmaceuticals currently available. Psychedelic compounds, such as psilocybin, may be a core part of the next generation of psychoactive medicinal treatments. The burden of mental illness has increased significantly over the past 25 years. As reported in the Global Burden of Diseases, Injuries, and Risk Factors Study (GBD) completed in 2019, the prevalence of mental illness increased 48.1% from an estimated 654.8 million cases in 1990 (95% UI 603.6-708.1) to 970.1 million cases in 2019 (900.9-1044.4) [1]. Although the methods used to estimate the worldwide prevalence of mental illness have been varied and thus the results inconsistent, measures such as years lived with disease (YLD) and disability-adjusted life years consistently rank mental illness as a top ten and often top five causes of global disease burden [1-3]. Further, the COVID 19 pandemic has been an unprecedented stressor, increasing the burden of mental illness both directly and indirectly. The full impact of the pandemic on mental health remains poorly understood and a topic of ongoing investigation [4,5]. In the US, stress-induced conditions like major depressive disorder, bipolar disorder, and generalized anxiety disorder were significant contributors to the increase in mental illness before the pandemic, and a recent review suggested that the point-prevalence of these disorders increased by 30% to 50% during the pandemic [6]. This is of significant concern since suicide rates in the US have already increased by 35% from 2000 to 2018 [7]. The wide overlap in symptoms between various disorders like major depression, bipolar I and II disorder, and generalized anxiety disorder are implicitly confounding. Specific manifestations with unique symptoms do not always characterize these illnesses and are also frequently comorbid, likely increasing the burden of mental illness is complex and perhaps unpredictable ways [8]. Further, the societal stigmas associated with mental illness remain stubbornly prevalent in the US, partially thwarting efforts to educate the general population, conduct research on new, novel treatments, and treat mental illness as an organic disease rather than as a character flaw [9]. The financial implications of mental illness are as severe as the human impacts: The World Health Organization estimates that depression and anxiety alone cost the world economy an alarming 1$ trillion US dollars each year [10]. There is a need for innovative treatments.

A historical account of psychopharmacology

A brief review of the historically significant advances in the treatment of mental illness with medication will serve to put the current research on psychedelic molecules into perspective. This short historical account includes the major past breakthroughs in developing medications to treat mental illness. Although limited success has been achieved, much failure has also occurred, which underscores the present need for new approaches that may include psychedelic drugs. The first antipsychotic medication, chlorpromazine, ushered in the field of psychopharmacology in the 1950s and began the process of understanding the underlying neuroscience of mental illness [11,12]. Although this medication was initially purposed as an augmentation for general anesthesia, a year after its introduction saw its first use as an antipsychotic [12]. The introduction of chlorpromazine and advances in brain imaging were foundational in the development of the serotonin/dopamine hypothesis of mental illness. Rather than describing a purely electrical phenomenon, in this model of mental illness, dysregulated levels of synaptic serotonin and/or dopamine are the underlying cause of mental illness. Although initial research focused on its anti-serotonergic properties, chlorpromazine is primarily a dopamine receptor antagonist, with a particular affinity for the D2 receptor. It was often used in state-run hospitals as the first medication capable of effectively treating severe mental illnesses such as schizophrenia. However, chlorpromazine also induces many unpleasant side effects such as dystonia, orthostatic hypotension, blurred vision, and other extrapyramidal effects [13]. Second-generation antipsychotics, beginning with the reintroduction of clozapine in 1990, have provided similar relief from the disabling psychoses associated with schizophrenia but with fewer extrapyramidal effects. Even these medications can cause severe, unwanted side effects such as metabolic disturbances and agranulocytosis, a potentially fatal complication [14-16]. Aripiprazole, one of the second-generation antipsychotics used for schizophrenia [17] is also often prescribed to treat bipolar I disorder [18,19]. The use of aripiprazole in the treatment of two disparate mental illnesses underscores the overlapping etiologies of mental illness. Second-generation antipsychotics have been the subject of randomly controlled trials as a treatment for a wide variety of illnesses including obsessive-compulsive disorder, personality disorders, substance abuse, Tourette’s syndrome, and post-traumatic stress disorder (PTSD) [20].

Antidepressant medications have also been in use for several decades including drugs in classes like the monoamine oxidase (MAO) inhibitors [21] and tricyclic antidepressants [22]. Although effective in the treatment of depression, these drugs often caused intolerable and occasionally life-threatening side effects that limited their utility [23]. For decades these were the main, and essentially only, pharmaceutical treatment options for depression until the next generation of mood disorder medications were developed.

An improvement in the medications used to treat mental illness, particularly major depression, occurred in the late 1980s with the release of fluoxetine (i.e., Prozac), the first US Food and Drug Administration (FDA)-approved selective serotonin reuptake inhibitor (SSRI) [24]. Fluoxetine was the beginning of a new era in the medical treatment of depression. In line with the still prevalent hypothesis of mental illness as an imbalance of neurotransmitters, its efficacy was attributed to an increase in the extracellular availability of serotonin by presynaptic blockade of the serotonin reuptake transporter (SERT) [25,26]. Fluoxetine and other SSRIs, as compared to placebo, generally improve depressive symptoms to varying degrees. However, the prevalence of undesirable side effects combined with a relatively modest effect size has generated questions as to their ultimate utility [27,28]. Unfortunately, the side effect profile of SSRIs often results in poor medication compliance [29]. Nevertheless, as a class, SSRIs were a significant improvement in the pharmacological treatment of mood disorders. Aside from the SSRIs, multiple non-SSRIs medications such as mirtazapine and bupropion are used to treat depression and a broad spectrum of other mental illnesses [30].

The prevalence and therapy of treatment-resistant depression

Antidepressants and anxiolytics have improved the treatment of depression and other mental illnesses over the past 50 years. Despite the development of multiple drug classes and the repurposing of older medications, the treatment of depression has remained enigmatic because the prevalence of mood disorders has not significantly waned during this time [31]. The underlying cause of this “treatment-prevalence paradox” is thought to be multifactorial. One such proposed reason is an initial overestimation of antidepressant medication efficacy combined with a lack of effect durability (a lessening of medication efficacy over time) [32]. Indeed, many medications aimed to treat mental illness have led to a diagnosis of a refractory disease that does not adequately respond to pharmacological treatment or becomes resistant to treatment at a previously effective dose of medication [33]. When treatment is ineffective, the most common response is to either increase the dose of the current medication or to change medications [34]. This has given rise to a common phenomenon where patients experience an early remission of symptoms only to suffer subsequent relapse or - as is often the case - fail to respond to any medication at all. These phenomena have led to a new diagnosable condition, treatment-resistant depression, which occurs in 30% of patients treated for major depressive disorder, and is defined as a failure to achieve disease remission after treatment with at least two first-line antidepressants [35]. Over time, treatment-resistant depression results in major depressive disorder [36].

Despite the seemingly intractable nature of its name, two therapeutic approaches are showing promise in treatment-resistant depression. The first, transcranial magnetic stimulation (TMS) has been available since 1985 [37]. It is a non-invasive technique that transmits a pulsed magnetic field deep into the brain, inducing electrical current modulation in neural circuitry [38]. The technology has improved considerably since its introduction and was FDA-approved in 2008 for treatment-resistant depression in several carefully controlled, double-blinded clinical trials [39,40]. Further, repetitive TMS sessions even produce improvement in geriatric patients, a subpopulation that has been notoriously difficult to treat [41]. The second approach for treatment-resistant depression relies on the drug, ketamine, which is a racemic mixture usually given intravenously. The S enantiomer, esketamine, is the more effective variant that binds to the N-methyl-D-aspartate (NMDA) glutamate receptor as an uncompetitive antagonist with higher affinity than the R enantiomer, arketamine. Infusion of R,S ketamine in severely depressed individuals, often treatment-resistant, shows very rapid antidepressive effects, sometimes in less than two hours [42]. With such drastic results, the FDA designated esketamine a breakthrough medication in 2013, and the clinical trials that ensued led to complete FDA approval for treatment-resistant depression in 2019 [43-45]. Esketamine can be delivered intranasally but must be administered in a clinical setting. Historically, ketamine has a long history as an anesthetic in veterinarian practice and at doses above 0.4-0.6, mg/kg is a dissociative anesthetic in humans with consciousness-altering effects similar to those of the classic psychedelics [42]. However, ketamine is not a “classic psychedelic” molecule because it is not an agonist for the 5-hydroxytryptamine 2A receptor (5-HT_2A_R), which is a receptor for the endogenous neurotransmitter serotonin [46]. This establishes esketamine as a different class of psychoactive medication than the classical psychedelic drugs (5-HT_2A_R agonists), a point that is often confused in the literature.

Psilocybin is a classic psychedelic drug that is therapeutic for mood disorders

**Figure 1:**
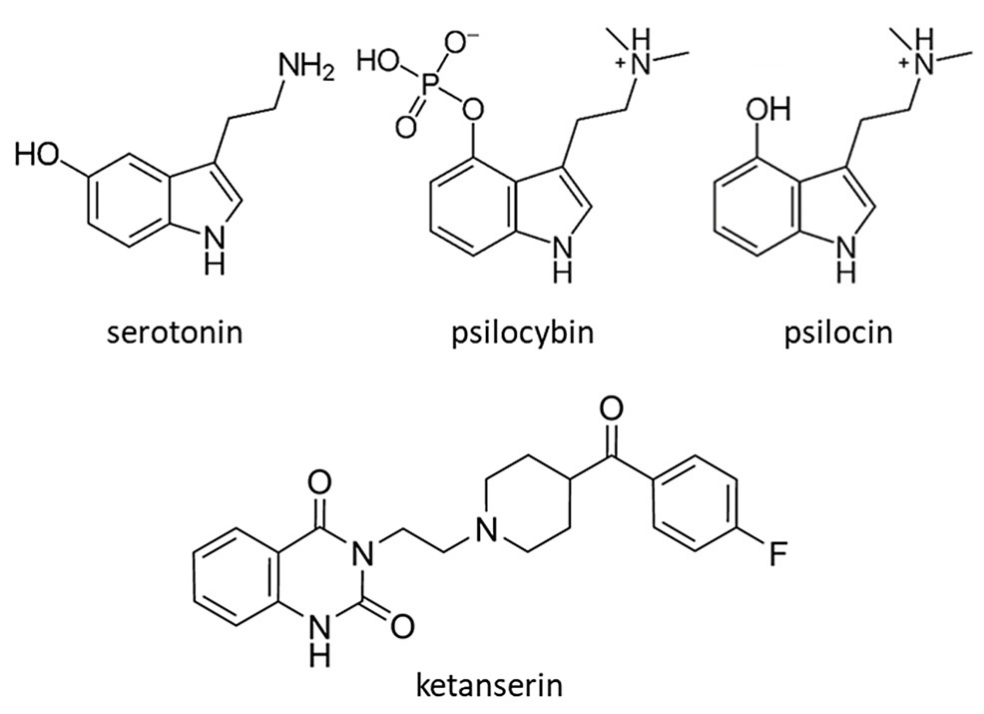
Molecular structures of serotonin, psilocybin, psilocin, and ketanserin. (Creative Commons CC BY-SA 3.0; Public Domain)

Recently, research on psychedelic molecules has increased substantially with a renewed interest in their potential as drugs to treat severe intractable mental illnesses. Colloquially the term “psychedelic” has multiple meanings, many of which are negative, with connotations including words such as hallucination, dissociation, altered states of consciousness, and mystical experience [47]. Many naturally occurring molecules such as psilocin, dimethyltryptamine (DMT), 5-methyl-O-DMT, mescaline, and d-lysergic acid amide (LSA), and synthetic molecules like lysergic acid diethylamide (LSD), 2,5-dimethoxy-4-iodoamphetamine (DOI), and 3,4 methylenedioxymethamphetamine (MDMA) are considered psychedelics because they stimulate altered states of consciousness. However, they vary in their receptor-specific mechanisms (nicely reviewed in [48]. The focus of this review is on psilocybin as it is a precursor to a classic psychedelic agonist for the 5-HT_2A_R (Figure 1). It is presently undergoing intense investigation as a pharmacotherapeutic for several mental illnesses. Psilocybin has also recently received breakthrough therapy status in 2018 from the FDA for the treatment of major depressive disorder through the private company, COMPASS Pathways [49]. In 2019, a non-profit research organization, Usona Institute, received similar approval for psilocybin [49]. Further, basic research into the mechanism(s) of action for psilocybin using a broad set of experimental methods is uncovering details of brain function, specifically unraveling the details of serotonergic pathways.

Psilocybin is the primary psychoactive molecule naturally found in over 150 species of mushrooms that grow throughout the world. This was verified in the late 1950s when the Swiss chemist Albert Hofman both synthesized and extracted psilocybin from the mushroom species *Psilocybe mexicana* [50]. Psilocybin is a pro-drug with a 4-O phosphate group that is removed by alkaline phosphatase or nonspecific esterases in the intestine and liver during first-pass metabolism to produce psilocin, its active metabolite [51]. Structurally, psilocin closely resembles the neurotransmitter serotonin and is therefore serotonergic (Figure 1). Psilocin, as a 5-HT_2A_R agonist, binds with a K_i_ of ~40 nM, [52], which is about 10-fold higher compared to serotonin’s binding affinity with a K_i_ of ~400 nM [53]. In general, higher affinity binding to the 5-HT_2A_R tends to produce stronger psychoactive effects for some compounds. For example, LSD binds to the receptor with a K_i_ of ~4 nM while a structurally related compound, lisuride, has a K_i_ of ~12 nM [54] and does not produce psychoactive effects like LSD [55]. The receptor affinity-dependent hallucinogenic effects of these compounds are further demonstrated by dose dependency: LSD induces hallucinations at doses of 100 µg/70 kg while a typical hallucinogenic dose of psilocybin is 30 mg/70 kg [56,57].

Similar to the dramatic increase in potency caused by the addition of a single methyl (methamphetamine) group to amphetamine, a remarkable change in physiological effect occurs from the addition of two aminomethyl groups and one indole hydroxyl group to serotonin, producing psilocin (Figure 1) [58]. Under normal conditions, serotonin does not produce hallucinations, dissociation, or altered states of consciousness like psilocin. Interestingly, when systemic serotonin levels are dysregulated in serotonin syndrome, which is generally a consequence of additive serotonergic drug interactions, altered mental states can occur including agitation, anxiety, disorientation, restlessness, and excitement [59].

Although progress has been made in elucidating the molecular mechanisms of psilocin, the full mechanism(s) is only partially understood. The structural similarity to serotonin would seem to make understanding the molecular events of psilocin more tractable, but even serotonin neurotransmission is complicated [60]. The molecular mechanisms of psilocin have recently been reviewed in more detail elsewhere [61,62]. To avoid redundancy, they will be briefly described here. The 5-HT_2A_R is a G protein-coupled-receptor that works with G_q_-like proteins, activating a variety of intracellular signaling cascades. Ligand binding to the 5-HT_2A_R activates a G_q_ protein followed by activation of the effector, phospholipase C-β. This leads to an intracellular increase in inositol 3-phosphate, which triggers an increase in cytoplasmic calcium. The increase in calcium activates protein kinase C and other protein kinases, producing additional downstream effects. Although the precise molecular pathways for the acute hallucinogenic effects of psilocin are unknown, the indirect involvement of dopamine and glutamatergic modulation of the α-amino-3-hydroxy-5-methyl-4-isoxazole propionic acid (AMPA) and N-methyl-d-aspartate (NMDA) receptors have been implicated. A downstream effect is an upregulation of brain-derived neurotrophic factor (BNDF), which is decreased in many forms of mental illness. BNDF also plays a major role in a diverse array of neuroplastic effects, including the insertion of AMPA receptors into the cell membrane at the synapse [63]. The influence of BDNF could in part explain the durable effects of psilocin (see below) long after it has been cleared from the body. Among other factors, the molecular efficacy of psilocin has been linked with its binding to additional 5-HT receptors [62].

Research on psychedelics has been moving at an exponential pace with over 2,400 publications in the last two years. Although the field is broad, emerging trends in publication content suggest a shift from basic science research in older publications to clinical trials more recently [64]. The increased interest in psilocybin is reflected in 401 publications since 2020. Although it is still a Schedule 1 drug, the renewed interest in psilocybin ironically comes from its potential as a therapeutic agent in a wide array of medically relevant problems, many of which have been intractable with current FDA-approved medications. Substance use disorder (opioid, nicotine, and alcohol), a wide array of anxiety disorders, treatment-resistant depression, fear of death in terminal disease, phantom pain, and many other conditions are all being studied with psilocybin therapy (reviewed in [49]).

A unique target of psilocybin therapy is its recent use to treat anxiety and depression associated with the end of life in patients with terminal diagnoses. Therapies combining SSRIs with methylphenidate (Ritalin), a stimulant used to treat ADHD, have had limited success in improving anxiety and depression in palliative care for terminally ill patients [65]. Due to the delayed onset of symptom relief for SSRI class drugs, they have not been tested for therapeutic efficacy in patients with depression and anxiety associated with imminent death [66]. The fear of death is common even in healthy individuals and is heightened when death is imminent, such as in cancer patients with a poor prognosis. It is unfortunate that the best pharmacological interventions to treat anxiety and depression work too slowly to be of benefit in this patient population (historically reviewed in [67]). Psilocybin, however, does not appear to suffer from a similar lag in the onset of therapeutic action. A double-blind, crossover, randomized control trial of cancer patients with comorbid anxiety and/or other mood disruption (n=51) were treated with a low (1-3 mg/70 kg) dose of psilocybin in the placebo-like condition or a single high (22 or 30 mg/70 kg) dose of psilocybin in the treatment condition [68]. Outcomes were measured using a battery of subjective questionnaires completed by both the participants and by community observers measuring changes in effect, behavior, and spirituality at baseline, five weeks after each dose condition, and at six months. The results suggested that a single high dose of psilocybin in a carefully controlled setting may substantially reduce depression and anxiety in terminally ill patients, with a durable effect even six months after treatment [68]. For example, 82.6% of participants reported significantly increased life satisfaction six months after receipt of the high-dose psilocybin. Interestingly, six months after receiving high dose psilocybin, 67.4% reported that the event (the receipt of psilocybin) was one of their top five most significant life experiences, remarkable since the average age of the participants was nearly 60 years (56.5). This study used a rigorous design, appropriate methods, and thorough statistical analysis of the data while adhering to established procedural details for well-controlled and safe experiments with psilocybin in human subjects - and may provide a model for future experimentation [69]. Similar results were observed in an independent study that also used psilocybin to treat death anxiety [70]. With remarkable outcomes, from original research of an ancient molecule, clinical trials for psilocybin-based therapy in treating a wider array of mental illnesses continue to gain momentum.

## Review

Due to a surge in the field of psychedelics, the recent literature contains several excellent reviews [48,62], particularly of psilocybin [49,71], and their role in treating mental illnesses. In this narrative review, we focus on studies in the last two years, attempting to illuminate and integrate: 1) the newest clinical trial data of psilocybin therapy; 2) studies on how psilocybin promotes neuritogenesis; 3) the relationship between the acute and long-term effects of psilocybin; 4) intrinsic brain network alterations after psilocybin treatment, and 5) the future of psilocybin therapy and research.

Phase 2 clinical trials are generally favorable for psilocybin therapy

Randomly controlled clinical trials are the gold standard in determining the efficacy of new therapeutic interventions for a disease or disorder in human subjects. Searching the clinical trials database (made available to the public in 2000; clinicaltrials.gov) with broad terms can provide a snapshot of the magnitude with which current health issues are being studied in this registry. To illustrate this point, a simple search for “cancer” retrieves 88,879 total studies with 37,673 completed and 29,335 at various stages of activity. The status of the remaining studies is either withdrawn, suspended, terminated, or unknown. Many cancer clinical trials progressed to phase 4 with 1,444 completed, or at various stages of activity. This reflects that the number of FDA-approved interventions for cancer treatment is large and retroactively being studied for continued efficacy and safety. Although it is impossible to determine an exact impact on patient outcomes by reviewing a database in this manner, it can be surmised that the treatments investigated in these studies extended or saved the lives of thousands.

Using an equally broad search term like “mental illness” in the clinical trials database retrieves 32,001 total studies, which underscores the magnitude of testing for therapeutic treatments. Of these total studies, 26,177 are completed or still at various stages of activity. Searching for “psilocybin” and “mental illness” currently retrieves 60 studies at various stages of activity, which is the most for any first-generation classic psychedelic drug. For example, “lysergic acid diethylamide” and “mental illness” yields six active or completed clinical trials. However, these numbers underrepresent psilocybin and LSD in the clinical trial database; when the search term “mental illness” is removed, the number of active studies on psilocybin and LSD increases to 103 and 26 trials, respectively. These search term omissions reveal that LSD is being studied for other disorders like cluster headaches (NCT03781128) and general neuroplasticity in healthy subjects (NCT05177419). Psilocybin is being studied as a potential therapeutic for several conditions like body dysmorphic disorder (NCT04656301); anorexia nervosa (NCT04052568); mild cognitive impairment including early Alzheimer’s disease (NCT04123314); burden and quality of life in post-treatment Lyme disease (NCT05305105); and even frontline clinician burnout during the COVID 19 pandemic (NCT05163496). Most of these studies are phase 2 clinical trials and may have components of mental illness involving serotonergic pathways as underlying causes.

Systematic reviews and meta-analyses have been recently conducted due to the abundance of clinical trials on psilocybin. Although many of these are broadly focused on psychedelics in general, two are focused on psilocybin. The first review centers on the safety profile of psilocybin as a drug and filtered through over 2,302 publications, distilling down to 44 referenced papers and eight used in the results, revealing some compelling data [72]. The safety profile for psilocybin is excellent, especially for psychoactive medication. First, the LD_50_ (dose that kills 50% of subjects) of psilocybin in mice is about 285 mg/kg [73], which extrapolates to about 22.1 g/kg for an average adult with a weight of 77.5 kg. Second, this allows the calculation of the therapeutic index (LD_50_/effective dose of psilocybin), a measurement of the “window” between the dose where a medication becomes clinically effective and the highest possible dose before it becomes dangerously toxic. For psilocybin, the therapeutic index is 3,683, using 0.006 g as an average minimum effective dose [73]. Using the same 77.5 kg average human weight, the therapeutic index is very narrow for heroin, 6; for alcohol, 10; and for cocaine, 15. For the previously discussed SSRI, fluoxetine, the therapeutic index is 100, suggesting that psilocybin maybe 40 times less likely to become toxic, and may be effective over a much wider range of doses [72]. Even with a moderately high dose of 20 mg, psilocybin is still over 10 times as safe as fluoxetine - a drug routinely prescribed many times a day. This underscores the potential safety of psilocybin as a therapeutic. A favorable safety profile for psilocybin was also suggested in a recent study that examined the adverse effects of psilocybin reported during clinical trials [74].

The second psilocybin-specific systematic review examines the efficacy of the drug at various doses in treating cancer-induced depression (which was termed secondary depression) or major depressive disorder (termed primary depression) [75]. After filtering 1,044 publications, the study focuses on five phase two clinical trials with quantitative meta-analysis; four are randomized control trials and one is an open-label trial. These include an aggregate of 136 participants (60 male, 76 female) with an average age of ~48.9 years. The trials use varying doses of psilocybin and study duration. The overall effect size in favor of psilocybin is 1.289 with a 95% confidence interval, (CI) = [1.020, 1.558] and heterogeneity I^2^ = 50.995%, p<0.001, suggesting cross-study variation of moderate impact. When subgroup analysis is performed for a dose range of 30-35 mg/70 kg, the effect size in favor of psilocybin increases to 3.059, 95% CI = [2.269, 3.849], p<0.001. Presently, this is the most rigorous analysis of the available psilocybin clinical trial data using the high standards of a systematic review. The authors concluded the current evidence suggests that psilocybin may rapidly improve the symptoms of depression in the studied population, and called for further research [75].

Although not included in these systematic reviews, several other clinical trials have been completed, with similar findings. A 12-month follow-up study conducted after the conclusion of an initial randomized, waiting-list controlled trial produced promising results [76,77]. A total of 24 participants aged 21-75 with major depressive disorder (MDD) having GRID-Hamilton depression (GRID-HAMD) scores ≥17 (severe depression) completed the initial trial. The participants were randomized and then spilt into two groups: 1) immediate treatment and 2) delayed treatment after an eight-week delay to serve as a control group. The initial treatment group was then given psilocybin in two sessions ­- a first, moderate dose of 20 mg/70kg and a second larger dose of 30 mg/kg one week later. One month after the conclusion of the trial, the results suggested that psilocybin produced a significant and durable decrease in depressive symptomatology. At the one-month interval, the immediate treatment group showed a decrease in GRID-HAMD scores from ~24 (very severe depression) to ~8 (0-7 is not depressed) [76,78]. The participants were then reassessed at three, six, and twelve-month intervals. At the 12-month interval, a 2.4-fold decrease in mean GRID-HAMD scores compared to baseline was reported, with no reports of adverse effects attributed to the treatment in the intervening months. These data suggest that the reduction in depressive symptomatology associated with psilocybin treatment of MDD is durable, potentially lasting up to a year after only a two-dose administration [77].

Another clinical trial compared the effects of escitalopram (a commonly prescribed SSRI) and psilocybin in participants suffering from MDD [79]. This was the first trial to directly compare the efficacy of psilocybin to a commonly prescribed SSRI for the treatment of MDD symptoms. The authors did not find a statistically significant difference between the two drugs, but these data suggest that even if psilocybin is not superior to a commonly prescribed SSRI, it is at least equally effective. The authors further reported that secondary outcomes (as measured by the BDI-1A, HAM-D-17, and MADRS scales) slightly favored psilocybin in general. The failure to detect a primary outcome difference between psilocybin and escitalopram may have been due to insufficient power derived from the small sample size (n=59) [79]. Thus far, the results of clinical trials evaluating psilocybin as a treatment for mental illness have been promising.

Psilocybin promotes neuritogenesis at the cellular level

Contemporary models of mental illness, particularly depression, are diverging from the classic neurotransmitter imbalance hypothesis. Instead, a model of dysregulated neuronal connectivity, with degeneration or atrophy, is emerging as the possible underlying pathology. Stress-induced mood disorders like depression, bipolar disorder, and anxiety all share functionally defective neuronal connections and overall disrupted synaptic architecture [80]. Supportive of this newer model, fast-acting antidepressants like ketamine restore synaptic connectivity, stimulating the formation and growth of dendritic spines [81]. Fluoxetine can also restore synaptic connectivity, but it requires weeks or months to do so with chronic, daily administration [80]. In contrast, in studies using immature cortical rat neurons in vitro, ketamine’s effect on dendritic spine growth does not require a continuous dose over long periods of time but can occur with treatment for less than one hour. LSD can also produce these effects, though ketamine and LSD bind to different receptors. Further, all psychedelic molecules tested including dimethyltryptamine (DMT), 2,5-dimethoxy-4-iodoamphetamine (DOI), and psilocin produces similar effects. These effects include increased dendritic arbor complexity as well as spine growth, which are components of neuritogenesis. Significantly, the effects of the tryptamine class of psychedelic molecules are blocked after pretreatment of the cells with ketanserin, a 5-HT_2A_R antagonist (Figure 1), likely demonstrating the involvement of this receptor [82]. The results of these cellular assays demonstrate a significant change in the morphological complexity of individual rat cortical neurons cultured in vitro. They may indicate that these changes take place in vivo as well, perhaps accounting for the observed efficacy of psychedelic compounds in the reduction of the symptoms of depression and anxiety.

In a mouse model expressing the green fluorescent protein (GFP) in a subset of layer 5 and 6 pyramidal cells, treatment with psilocybin stimulates neuritogenesis in the prefrontal cortex in vivo [83]. With a two-photon microscopic system, imaging intermittently for long periods of time in the medial frontal cortex, dendritic spine formation occurs after a single 1 mg/kg intraperitoneal dose of psilocybin. This system allowed long-term tracking of 1,820 spines from 161 neuronal branches in 12 animals. New spine growth appeared relatively fast (within one day). The number of spines increases ~7% after day 1 and ~12% after day 7, which is the period for the greatest effect after treatment. The durable change was again noted, as a subset of the spines persisted for up to one month after the single treatment with psilocybin. Electrophysiological experiments demonstrated that an increase in excitability also occurs, suggesting that these new dendritic spines increase functional neurotransmission. The authors also evaluated the effect of psilocybin on a stress-induced learned response in mice, noting that although the cross-animal effect size was not significant, all but one animal either stayed the same or reduced the number of failures to escape from a foot-shock stressor. This experiment used a murine learned helplessness condition to model the human stress response, and the results suggested that a single dose of psilocybin may improve the downstream sequelae associated with chronic stressors. The authors concluded that in a murine in vivo model, psilocybin induced dendritic spine growth, enhanced neuroplasticity, and thus may increase or restore the number of neuronal microcircuitry connections in the frontal cortex [83]. If depression is indeed a result of the loss of neuronal connections in the frontal cortex, it is possible to suggest that the therapeutic effects of psilocybin in human clinical trials may in part be due to long-lasting neuritogenesis in the brain.

Durable effects vs the acute hallucinogenic mystical effects of psilocybin

The ability to induce altered states of consciousness defines psychedelic drugs. If psilocybin is to become a first-line treatment for mental illness, the following question must be answered: Are the acute hallucinogenic and mystical effects of psilocybin required as a prelude to promote and stimulate the durable effects of increased synaptic connectivity via neuritogenesis or can the consciousness-altering effects of the drug be blunted while retaining the neuritogenic properties of psilocybin? Griffiths et al. [84] performed a dose-effect study and concluded that a positive, linear, dose-dependent relationship existed between the magnitude of the mystical experience reported by participants after ingestion of a single dose of psilocybin and subsequent long-term improvements in mood. Hirschfield et al. [85] completed a recent meta-analysis and found robust evidence supporting a direct correlation between dose and intensity of experience, but cautioned that these data could not be generalized to unsupervised use of psilocybin due to unpredictable interactions between setting and drug response. This warning suggests that psilocybin may need to be administered under controlled, clinical conditions until a better understanding of the interactions between setting, subject, and dosage is found. Anecdotal evidence has suggested that using psilocybin delivered in low doses, colloquially “microdosing,” may bypass the mystical/hallucinatory experiences associated with higher doses of the drug while preserving long-term mental health improvements. However, when studied in a randomly controlled trial against a placebo, this effect has not been replicated [86]. As noted above, If the undesirable acute mystical/hallucinatory experience cannot be separated from the desirable long-lasting mental health benefits, then the medication will only be administrable under tightly controlled, supervised conditions. This would limit the utility of psilocybin as a first-line treatment for mental illness.

The synthesis of new molecules based on the structure of known psychedelic drugs shows promise in decoupling the mystical experience from the synaptic plasticity effects. These “psychoplastogens” are small molecules that induce neuritogenesis in vitro, yet are predicted to not produce hallucinations in vivo [87]. Proof-of-concept experiments have been successful at structurally transforming ibogaine to tabernanthalog (TBG) [88] and DMT to isoDMT [89]. Both TBG and isoDMT promote neuroplasticity and have anti-addictive and behavioral effects, respectively. Further, both TBG and isoDMT do not induce a head-twitch response in rodents, which correlates well with the hallucinogenic properties of true psychedelics like LSD and psilocin [90]. Recently, the same research group at UC Davis used a 5-HT_2A_R-GFP construct as a biosensor (psycheLight), to discover a molecule, AAZ-A-154, that resembles DMT and does not induce a head-twitch response in mice while still promoting neuritogenesis in vitro [91]. The investigation of these “psychoplastogens” has produced early promising results, suggesting that even if naturally occurring psychedelics like psilocybin resist the separation of hallucinogenic properties from neuritogenesis, it may be possible to build a synthetic substitute with this characteristic. So far though, these novel compounds have only been tested in vitro and in murine models in vivo. Human clinical trials remain a future consideration but until these novel new agents can undergo rigorous testing, it is not possible to assess their potential as a treatment for mental illness.

Psilocybin promotes brain network integration at the organismal level

The experimental confirmation that psilocybin induces neuritogenesis in the mouse brain suggests the interesting inference that psilocybin might also promote or stimulate connectivity in higher-order intrinsic brain circuits and/or networks. If this is true, then the effect may offer insights into its efficacy in treating mental illnesses such as depression in humans. Depression, and specifically MDD, is the result of disconnections not only at the cellular level but also at the level of brain intrinsic networks [92]. Neuronal synaptic weakening or complete disconnections at the cellular level may eventually result in this network-level atrophy. Brain network-level dysconnectivity may also explain the modular thinking often seen in patients with depression. They frequently become fixated on negative thought patterns that strengthen their depressed condition. This ruminative thinking pattern tends to be self-reinforcing over time in MDD and results in increased functional connectivity between (and within) the default-mode network, DMN (involved in self-referential processing), and the subgenual prefrontal cortex, sgPFC (involved in emotional regulation and reward) [93]. Over time, the increased functional connectivity within the DMN and to the sgPFC is suggestive of the rigid, negative thinking that is symptomatic of MDD. This increasedbrain network-level connectivity may result in connectivity decreasesfor other functional brain networks or, at minimum, dampen their activity [94]. This would be predicted to increase modularity within the DMN and between the DMN and sgPFC, giving rise to less integration with other intrinsic brain networks.

Psilocybin’s acute effects during the psychedelic state lead to increased brain network connectivity in individuals without underlying mental illness [95]. This temporary “rewiring” is extensive and suggests greater integration across dozens of brain network interfaces and while some of the effects are temporary, some of the network alterations endure for at least a month after psilocybin ingestion [96]. These observed phenomena suggest that therapy with psilocybin may result in functional improvements in brain networks thought to be deteriorated in patients diagnosed with MDD.

Recent work supports this hypothesis. Using functional magnetic resonance imaging (fMRI), one study found that psilocybin may reduce the hyperconnectivity in the DMN thought to be the result of depression, and consequently enhance connectivity in other brain functional networks, leading to a more “globally” integrated brain [97]. The study was an extension of previous work [79,98] and used two groups; one diagnosed with treatment-resistant depression (TRD) and a second diagnosed with MDD. The 19 patients in the TRD group participated in an open-label trial, assessing changes in fMRI from baseline after the administration of two doses of psilocybin (10 and 25 mg), given one week apart. This arm of the study was designed to evaluate the rapid antidepressant effects of psilocybin. In addition to pre- and post-administration fMRI, the participants in the TRD group were subject to remote clinical follow-up a week, three months, and six months post-treatment. A double-blind randomized control trial (DB-RDT) was used for the MDD group, comparing the efficacy of psilocybin to escitalopram (similar to the work of Carhart-Harris et al., [79] noted above). However, this DB-RCT also attempted to parse a possible mechanism of action by examining pre- and post-administration fMRI results. Fifty-nine patients were included in the study: 30 in the psilocybin treatment arm and 29 in the escitalopram arm. The study was blinded by informing the participants that they would all receive varying doses of psilocybin, implying that the trial was evaluating the efficacy of a sliding scale of doses and not two discreet drugs. The psilocybin group received two 25 mg doses three weeks apart as well as a daily placebo (microcrystalline cellulose) for the first three weeks followed by two placebo capsules for the next three weeks. This was designed to simulate the dosing escalation necessary for the escitalopram arm. The escitalopram group received 1 mg doses of psilocybin (likely sub-clinical in effect) three weeks apart, but then received one 10 mg capsule of escitalopram per day for the first three weeks and two 10 mg capsules per day for the second three weeks. Neuroimaging with fMRI was conducted after the six-week DB-RCT.

The authors used two sets of outcome measures: 1) Depression measurements using the Beck Depression Inventory (BDI-1A) and 2) Comparison of pre- and post-trial fMRI. In the TRD group, baseline BDI scores were ~35 (severe depression) but dropped ~21 points (~14 is viewed as mild mood disturbance) after psilocybin treatment, suggesting a significant decrease in depressive symptomatology. Baseline BDIs in the DB-RCT MDD group were lower by about ~6 points than in the TRD group; BDI results were designated as a secondary outcome for this arm of the trial. In this study arm, however, statistically significant changes in BDI scores were found at the two-, four-, and six-week time points with participants in the psilocybin group scoring 8.73 points lower at two weeks, 7.79 points lower at four weeks, and 8.78 points lower at six weeks. As opposed to the indeterminate results reported by Carhart-Harris et al. [79], these data suggest that psilocybin may be superior to escitalopram in reducing the symptoms associated with MDD. Analysis of pre- and post-trial fMRI suggested a possible mechanism for the observed outcome. In the psilocybin groups, brain modularity decreased in the DMN, with consequent increases in connectivity to the intrinsic executive network (EN) and intrinsic salient network (SN), which may indicate a more complete integration of brain intrinsic networks. The fMRI changes appeared quickly in participants treated with psilocybin, with measurable alterations in brain connectivity after only 24 hours. The psilocybin-induced brain network changes correlated well with the decreases in BDI score. Interestingly, increased global brain network connectivity, suggesting better integration, was not observed in the escitalopram treatment group [97]. This study is of increased interest because it is the first RCT with psilocybin against a previously studied antidepressant medication that reported greater treatment efficacy for the psychedelic while also suggesting a possible mechanistic explanation for the observed outcome differences.

Psilocybin in the near future after a difficult past

Mental illness is a complex set of diseases. Definitive diagnosis of specific disorders is difficult because simple biomarkers do not exist to test for changes in blood levels despite the extensive search for them [99]. At present, qualitative measures continue to guide diagnosis and therapeutic treatment with medication even without precise causes. However, the mystifying etiologies of mental illness have slowly started to be revealed over the last 75-85 years. When LSD was synthesized in 1938, the psychedelic era formally began; informally, it had been underway for millennia under the guise of psilocybin, which we could not recognize as a psychedelic until the 1950s. As early as 1950, LSD was being studied to help with psychotherapy [100] and psilocybin was studied to treat convulsions in 1959 [101]. During that time, the structure and function of DNA were barely understood let alone the intricate neurotransmitter functions of serotonin. The social and political constraints that stopped psychedelic drug research efforts in the 1960s and early 1970s, leading to the eventual classification of these compounds as Schedule 1 drugs, were a result of much scientific ignorance. However, technological development did not stop in the 1970s and we can now understand biology at the atomic level due to studies using sophisticated methods that were unimaginable in the 1970s. Ironically, our continued understanding of biological processes at fundamental levels has allowed a more complete recognition of the many manifestations of mental illness. For example, what was once called “manic depression” is now classified as bipolar disorder 1 or 2. Although psychiatry is now a rich, evidenced-based medical specialty firmly rooted in psychopharmacology, past and current FDA-approved medications (save esketamine) are losing overall efficacy in treating increasingly prevalent mental illnesses.

The hallucinogenic properties of classic psychedelic drugs like psilocybin and its Schedule 1 designation have been a barrier to broad acceptance of its potential therapeutic effects in the US. For example, the National Institutes of Health (NIH) did not fund any grants specifically involving psychedelic clinical trials from 2006 to 2020 [102]. This period encompasses many successful government-funded clinical trials worldwide, including in Australia, Canada, Israel, New Zealand, and the United Kingdom, that have supported this research field [103-105]. As of this writing, the NIH has now funded a joint study between Johns Hopkins University, the University of Alabama, and New York University to study psilocybin as a therapeutic intervention for tobacco use disorder - the first US federal grant in 50 years for psychedelic treatment [106]. However, the lack of prior federal funding support has not stopped research in this area as over 15 centers dedicated to psychedelic research exist worldwide in the academic and private sectors. Moreover, nearly 50 publicly traded and nearly 40 privately held companies are investing heavily in the promise of psychedelics as therapeutics [107]. This sector has largely fueled the funding for psychedelic research over these last 5-10 years rather than the federal government (at least in the US).

Further research needs to be performed before psilocybin might be granted FDA approval for the treatment of mental illnesses like depression. Its polypharmacology makes understanding mechanisms of action difficult. Many receptors, beyond the 5-HT_2A_R, are potentially activated making psilocin’s molecular pharmacology complex [108]. The recent determination of the 3D structure for the activated 5-HT_2A_R bound to LSD will serve as a tool to help guide the search for more specific drugs [109]. The “psychoplastogen” class of molecules like TBG and isoDMT need to be studied in clinical trials to test the hypothesis that hallucinations are not induced and that they are efficacious in treating mental illness in humans. Phase 3 clinical trials of psilocybin should be done for the treatment of depression, taking into considerations that address a number of practical issues toward the possible use in psychiatry [110]. Additional phase 2 trials should be performed for the treatment of other mental illness disorders.

A far less tractable issue is the high degree of polymorphism that exists in the *HT2AR* gene [62,111]. The variations in alleles result in many single amino acid changes in the 5-HT_2A_R that affect ligand binding, which could attenuate the therapeutic effects of future drugs. This may partly explain the varied responses of individuals not only to psilocybin but also to other drugs that target this receptor. A likely future scenario might include a variety of structurally similar analogs to psilocybin for therapy that may cover the range of genotypes in the human population. The variability of the *HT2AR* gene serves as a reminder that in the pharmacotherapy of mental illness, a diverse array of treatment modalities is required. Thus, a variety of new therapies including TMS, ketamine, and perhaps psychedelics or psychedelic-like drugs will be used to decrease the prevalence of mental illness in the future.

## Conclusions

The renewed interest in psychedelic molecules as therapeutic tools for the treatment of mental illness has arisen from a deeper understanding of pharmacological mechanisms and neuroscience at the cellular, molecular, and brain network levels. In just 10 years, we are moving away from the notion of defective neurotransmitter concentrations at synapses as an underlying etiology. Instead, a newer hypothesis views defective neuronal synaptic connectivity as the primary etiology of mental illness. Studies with psychedelic molecules have greatly accelerated this contemporary model of mental illness and psilocybin is leading the way as a therapeutic intervention. Current evidence suggests that one or two doses of psilocybin can result in a significant reduction in depressive symptomatology, perhaps by a mechanism of induced neuritogenesis, leading to increased brain network integration. In short, psilocybin may heal the damaged brain. Should these findings be repeated in future studies and more clinical trials, FDA approval of psilocybin as a treatment for mental illness may: 1) help reduce the prevalence of mental illness and 2) ease the suffering of patients who have experienced repeated treatment failures with the best medications available today.
